# Disrupted Interhemispheric Synchrony in Default Mode Network Underlying the Impairment of Cognitive Flexibility in Late-Onset Depression

**DOI:** 10.3389/fnagi.2016.00230

**Published:** 2016-09-27

**Authors:** Zhenghua Hou, Yuxiu Sui, Xiaopeng Song, Yonggui Yuan

**Affiliations:** ^1^Department of Psychosomatics and Psychiatry, Institute of Psychosomatics, Zhongda Hospital, Medical School of Southeast UniversityNanjing, China; ^2^Department of Psychiatry, Affiliated Nanjing Brain Hospital of Nanjing Medical UniversityNanjing, China; ^3^Department of Biomedical Engineering, College of Engineering, Peking UniversityBeijing, China

**Keywords:** late-onset depression, voxel-mirrored homotopic connectivity, functional magnetic resonance imaging, cognitive function, cerebellum

## Abstract

The intuitive association between cognitive impairment and aberrant functional activity in the brain network has prompted interest in exploring the role of functional connectivity in late-onset depression (LOD). The relationship of altered voxel-mirrored homotopic connectivity (VMHC) and cognitive dysfunction in LOD is not yet well understood. This study was designed to examine the implicit relationship between the disruption of interhemispheric functional coordination and cognitive impairment in LOD. LOD patients (*N* = 31) and matched healthy controls (HCs; *N* = 37) underwent neuropsychological tests and functional magnetic resonance imaging (fMRI) in this study. The intergroup difference of interhemispheric coordination was determined by calculating VMHC value in the whole brain. The neuro-behavioral relevancy approach was applied to explore the association between disrupted VMHC and cognitive measures. Receiver operating characteristic (ROC) curve analysis was used to determine the capability of disrupted regional VMHC to distinguish LOD. Compared to the HC group, significantly attenuated VMHC in the superior frontal gyrus (SFG), superior temporal gyrus (STG), posterior cerebellar lobe (CePL) and post- and precentral gyri were observed in the bilateral brain of LOD patients. The interhemispheric asynchrony in bilateral CePLs was positively correlated with the performance of trail making test B (TMT-B) in LOD patients (*r* = 0.367, *P* = 0.040). ROC analysis revealed that regions with abnormal VMHC could efficiently distinguish LOD from HCs (Area Under Curve [AUC] = 0.90, *P* < 0.001). Altered linkage patterns of intrinsic homotopic connectivity and impaired cognitive flexibility was first investigated in LOD, and it would provide a novel clue for revealing the neural substrates underlying cognitive impairment in LOD.

## Introduction

Geriatric depression is a prevalent and disabling mental disorder in elderly people, often characterized by impaired cognitive function and emotional symptoms (Sheline et al., 2006; Unützer and Park, 2012). A recent longitudinal study revealed that 61% of baseline depressed patients suffered from a chronic disease duration of depression during 2 years’ of follow-up (Comijs et al., 2015). Furthermore, impaired visuospatial ability, information-processing speed, and delayed memory were persistent despite the remission of depressive symptoms after 1 year (Bhalla et al., 2006). Emerging research proposed that late-life depression (Koenig et al., 2014), especially late-onset depression (LOD), exhibit more dementia-related neuropathology, which could be regarded as an important risk factor for deterioration in Alzheimer’s disease (Sierksma et al., 2010; Dillon et al., 2014; Sachs-Ericsson et al., 2014). The problem with the medication of LOD is the lack of specific, objective biomarkers to assist clinicians in establishing an individualized diagnosis and in improving specific treatment. Currently, the neural underpinnings of LOD and progressive cognitive deterioration remain poorly characterized.

In the past decade, morphological and functional magnetic resonance imaging (fMRI) studies (Chen et al., 2012; Hahn et al., 2015; Lebedeva et al., 2015) of LOD have elegantly described the abnormalities in structure and function within many different brain regions, particularly in the cognitive control network (CCN) and the default-mode network (DMN), although inconsistent results were reported. Lim et al. (2012) reported that LOD patients exhibit reduced hippocampal volumes and memory function-related cortical thickness in the superior temporal cortex, anterior/posterior cingulate cortex and dorsolateral prefrontal cortex. Our previous work also found altered amygdala, hippocampal or cortico-cerebellar functional network in resting-state fMRI (RS-fMRI) and cognitive deficits in LOD (Yue et al., 2013; Yin et al., 2015a,b). Importantly, the cognitive dysfunction and abnormalities of DMN or hippocampal sub-regional networks persisted in LOD patients even though the depressive symptoms were remitted (Wang et al., 2012, 2015c; Shu et al., 2014). Evidence that resting state networks are disrupted in LOD has invigorated attempts to understand better putative markers of illness and cognitive deficit. Illustrating the neural underpinnings of cognitive impairment in LOD is essential in prompting research on early prediction, prospective clinical identification and optimal treatment targets. However, to date, few studies have elucidated the potential neuropathological mechanisms of cognition impairment in LOD.

Specifically, the disrupted information integrities between bilateral brains play a critical role in the pathophysiology of cognitive dysfunction (Zuo et al., 2010; Kourtidou et al., 2013; Wang et al., 2015b). Recent research has detected stronger amygdalar interhemispheric connectivity (Irwin et al., 2004) and decreased corpus callosum volume (Hahn et al., 2015) involving inter-hemispheric communication and cognitive function in depression. Utilizing the approach of voxel-mirrored homotopic connectivity (VMHC), which reflects the correlation of the synchrony of spontaneous brain functional activities between symmetrical regions in bilaterally hemispheric architecture (Salvador et al., 2005), researchers identified abnormal VMHC in several regions that were associated with clinical features in major depressive disorder (MDD; Lai and Wu, 2014; Wei et al., 2014). Specifically, Hermesdorf et al. (2016) detected interhemispheric asynchrony in DMN regions (e.g., precuneus, insula, etc.) in MDD. Meanwhile, recent studies and our previous results suggested that the changed network processes in DMN were related to the impairment of cognitive performance, and the balance in DMN, CCN and salience network processes are essential to maintain optimal cognitive function (Wu et al., 2013; Beason-Held et al., 2016). It is feasible to study the potential correlation between VMHC in DMN and cognitive dysfunction, for it provides a new perspective for understanding the role of DMN in the process of cognitive decline in LOD. However, to our knowledge, there are no studies investigating the possible relationship between the altered interhemispheric coordination in DMN and cognitive impairment in LOD patients.

The purpose of the present study was to explore the intrinsic difference of interhemispheric coordination between LOD patients and healthy subjects. Based on the abovementioned results, we sought to determine if regional disruption of VMHC in the prefrontal cortex, temporal gyrus, cerebellum or other DMN regions might exert an adverse impact on cognitive function. We hypothesized that the disrupted VMHC in regions of DMN would generate synergetic effects in the pathogenesis of cognitive dysfunction in LOD patients. Furthermore, we identified the performance of aberrant patterns of homotopic connectivity in distinguishing LOD patients from healthy controls (HCs).

## Materials and Methods

### Participants

All participants were recruited from the Affiliated Brain Hospital of Nanjing Medical University, China. All subjects were interviewed in a semi-structured interview included in the Structured Clinical Interview for DSM-IV Axis I Disorders (SCID-I/P), Clinician Version (First et al., 1997), and the diagnoses were determined by a consensus of at least two trained and senior psychiatrists. All participants also underwent diagnostic evaluations including clinical interview, review of medical history and demographic inventory. The participants met the following inclusion criteria: (1) they met the major depressive disorder in DSM-IV criteria at the time point of enrollment; (2) they were in their first depressive episode and the age of onset was over 55 years; (3) 17 items Hamilton Depression Rating Scale (HAMD-17; Hamilton, 1960) were greater than 17; (4) absence of another major psychiatric illness, including substance abuse or dependence; (5) absence of primary neurological illness, including dementia or stroke; (6) absence of severe medical illness that impair cognitive function obviously; (7) no history of receiving electroconvulsive therapy; (8) T2-weighted MRI of all patients did not show severe white matter impairment such as infarction or other vascular lesions; (9) have no psychotic symptoms (i.e., hallucination/bizarre delusions/thought broadcasting); (10) without taking antidepressant and within 6 months prior to the MRI scan. The inclusion criteria for HCs are similar to LOD patients except, meeting the diagnostic criteria for MDD and HAMD-17 score which was greater than 17, and with no first-degree family history of psychiatric illness. All subjects were unequivocally and naturally right-handed. Thirty-one LOD patients and 37 HCs were recruited in this study. The Nanjing Medical University Research Ethics Committee approved the study and written informed consent was obtained from all participants.

### Neuropsychological Measurements

All subjects underwent diagnostic evaluations, including the HAMD, and cognitive function testing with a neuropsychological battery that consisted of the Mini-Mental State Examination (MMSE), the Auditory Verbal Learning Test (AVLT)-delayed recall, the Digit Span Test (DST-forward and backward), the Symbol Digit Modalities Test (SDMT), the Verbal Fluency Test (VFT-animal and verb) and the Trail Making Test (TMT-A and B).

### Image Acquisition and Processing

All subjects underwent the MRI scans at the Affiliated Nanjing Brain Hospital of Nanjing Medical University. The subjects were scanned using a Siemens 3.0 Tesla scanner with a homogeneous birdcage head coil. Subjects lay supine with the head snugly fixed by a belt and foam pads to minimize head motion. A gradient-recalled echo-planar imaging (GRE-EPI) pulse sequence was set up to acquire resting-state images. The acquisition parameters of RS-fMRI were as follows: repetition time = 2000 ms; echo time = 30 ms; flip angle = 90°; acquisition matrix = 64 × 64; field of view = 240 mm^2^ × 240 mm^2^; thickness = 4.0 mm; gap = 0 mm; 31 axial slices and 3.75 mm^2^ × 3.75 mm^2^ in-plane resolution parallel to the anterior commissure-posterior commissure line. High-resolution T1-weighted axial images covering the whole brain were acquired utilizing a 3-dimensional inversion recovery prepared fast spoiled gradient echo (SPGR) sequence presented as follows: repetition time = 1900 ms; echo time = 2.48 ms; flip angle = 9°; acquisition matrix = 256 × 96; field of view = 250 mm × 200 mm; thickness = 1.0 mm; gap = 0 mm. Those above acquisition sequences generated 140 volumes in 7 min 6 s and 128 slices in 4.3 min, respectively. All subjects were guided to keep their eyes closed and to relax, to remain awake and not to think anything specific during scanning.

### Functional Image Processing

Functional images were preprocessed utilizing the Data Processing Assistant for Resting-State Function (DPARSF 2.3 Advanced edition) MR Imaging toolkit (Yan and Zang, 2010), which synthesizes procedures based on the Resting-State Functional MR imaging toolkit (REST^1^; Song et al., 2011), and statistical parametric mapping software package (SPM8^2^). The first 10 time points were discounted in order to ensure stable-state longitudinal magnetization and adaptation to inherent scanner noise. The remaining 130 RS-fMRI images were sequentially performed according to the following steps: (1) slice timed with the 31st slice as a reference slice; corrected for temporal differences and head motion correction (participants with head motion of more than 1.5 mm of maximum displacement in any direction (*x*, *y*, or *z*) or 1.5° of angular motion were excluded from the present study); (2) coregistered T1 to functional image and then reoriented; (3) for spatial normalization, T1-weighted anatomic images were segmented into white matter, gray matter and cerebrospinal fluid, and then normalized to the Montreal Neurological Institute space by using the transformation parameters estimated using a unified segmentation algorithm (Ashburner and Friston, 2005). The above transformation parameters were applied to the functional images and then the functional images with isotropic voxels of 3 mm resampled; (4) spatial smoothing undertaken with a 6 mm full-width at half-maximum isotropic Gaussian kernel; (5) the linear trend within each voxel’s time series removed; (6) nuisance signals (white matter, cerebrospinal fluid signals, head-motion parameters calculated by rigid body six correction) and spike regressors regressed out; (7) temporal bandpass (0.01–0.08 Hz) to minimize low-frequency drift and high-frequency noise filtered.

### Voxel-Mirrored Homotopic Connectivity

For the calculation of VMHC value in the geometric configuration of the cerebral hemispheres, the preprocessed functional images were transformed to a symmetric space conforming to the following procedure: (1) generated a mean image by averaging the normalized gray matter images for all subjects; (2) averaged the above mean image with bilateral mirrored version to create a group-specific symmetrical template; (3) registered every individual normalized gray matter image to the generated symmetric template, and then transformed the functional images based on the nonlinear strategy.

Subsequently, Pearson’s correlation analysis was conducted between each pair of time series within symmetrical interhemispheric voxels. The computed correlation coefficients were Fisher z-transformed to obtain a VMHC z-map for statistical analyses. For the clusters with significant differences, mean VMHC values were extracted for further analyses. The details of VMHC acquisition have been elucidated in a previous study (Zuo et al., 2010). In order to verify the reproducibility and robustness of the VMHC differences between LOD patients and HCs in the present study, the split-half validation approach was adopted. We randomly selected *N* = 16 LOD patients and *N* = 18 HCs from the initial sample and then performed the two-sample *t*-tests with age, gender, education and GMV as covariates.

### Statistical Analyses

Independent-sample *t*-test, Chi-squared test or Analysis of covariance (Statistical Package for the Social Sciences software, SPSS17.0, Chicago, IL, USA) was used to determine significant differences in demographic data, HAMD scores and neuropsychological performance between LOD and HCs. The Cohen’s *d* values that reflect the effective size of the statistical difference between groups was calculated by G*Power software 3.1 (Faul et al., 2007). For comparing of imaging data, the whole brain gray matter volume, age, gender and education level were used as covariates, the between-group differences of VMHC were calculated in REST and the results were multiple corrected with AlphaSim analysis as determined by Monte-Carlo simulation (threshold has been set at *P* < 0.001, corrected with voxel-level *P* < 0.01, cluster size: ≥55 voxels, see Analysis of Functional NeuroImages [AFNI]^3^) within the unilateral hemisphere of the symmetric template. The continuous variables are presented as mean ± Standard Deviation (SD). The relationships between VMHC changes and characteristic of depression in patients were examined by bivariate Pearson’s correlation analysis. We calculated the predictive performance of the altered VMHC values using sensitivity, specificity and area under the receiver operating characteristic (ROC) curves (Area Under Curve [AUC]: 0.9–1.0 = excellent; 0.8–0.9 = well; 0.7–0.8 = fair; 0.6–0.7 = poor; 0.5–0.6 = fail). Optimal cut-off between sensitivity and specificity were determined by maximizing the Youden’s index *J* (*J* = sensitivity + specificity − 1). Binary Logistic regression analysis was performed to integrate the combined distinguishing effect of brain regions with altered VMHC. The threshold of statistical significance was defined as *P* < 0.05.

## Results

### Demographic and Neuropsychological Data

Demographic and clinical characteristics of LOD and HCs were showed in Table 1, there were significant differences (*P* < 0.005) in years of education and cognitive performance but not in gender distribution (*χ*^2^ = 2.12, *P* = 0.15) and age (*t* = 1.62, *P* = 0.11). Compared to HCs, LOD group exhibited poor cognitive performance in extensive domains. As an additional statistical control, we presented results by controlling education level, age and gender.

**Table 1:** **Demographic and clinical characteristics of LOD and HCs**.

Items	LOD (*n* = 31)	HCs (*n* = 37)	Cohen’s *d*	Statistic value	*P* value
Gender (male: female)	10:21	18:19	/	χ^2^ = 2.12, df = 1	0.15^a^
Age (years)	68.00 ± 6.09	65.27 ± 7.52	/	*t* = 1.62, df = 66	0.11^b^
Education level (years)	9.52 ± 4.21	12.84 ± 3.04	0.90	*t* = −3.66, df = 66	0.000^b^
HAMD	31.42 ± 4.44	2.03 ± 2.33	8.29	*F* = 602.42, df = 2	0.000^c^
MMSE	28.23 ± 1.94	29.46 ± 0.93	0.81	*F* = 6.62, df = 2	0.002^c^
SDMT	17.16 ± 5.64	38.70 ± 10.16	1.50	*F* = 65.34, df = 2	0.000^c^
AVLT-delayed recall	10.84 ± 0.82	11.57 ± 0.69	0.96	*F* = 8.37, df = 2	0.001^c^
VFT	25.35 ± 6.66	39.00 ± 8.46	1.79	*F* = 27.66, df = 2	0.000^c^
DST	10.52 ± 1.55	13.54 ± 1.79	1.80	*F* = 32.73, df = 2	0.000^c^
TMT-A (s)	107.86 ± 30.78	74.59 ± 22.38	1.24	*F* = 21.68, df = 2	0.000^c^
TMT-B (s)	183.31 ± 49.89	144.27 ± 45.59	0.82	*F* = 14.90, df = 2	0.001^c^

*Abbreviation: LOD, late-onset depression; HCs, healthy controls; HAMD, Hamilton Depression Scale; MMSE, Mini Mental State Exam; SDMT, Symbol Digit Modalities Test; AVLT-delayed recall, Auditory Verbal Learning Test-delayed recall; VFT, Verbal Fluency Test-animal and verb; DST, Digit Span Test-forward and backward; TMT-A, Trail Making Test-A; TMT-B, Trail Making Test-B. Parametric values are represented as the Mean ± SD (standard deviation). ^a^Chi-square test. ^b^Independent-samples t-test. ^c^Analysis of covariance*.

### Voxel-Mirrored Homotopic Connectivity Data

**Figure 1:**
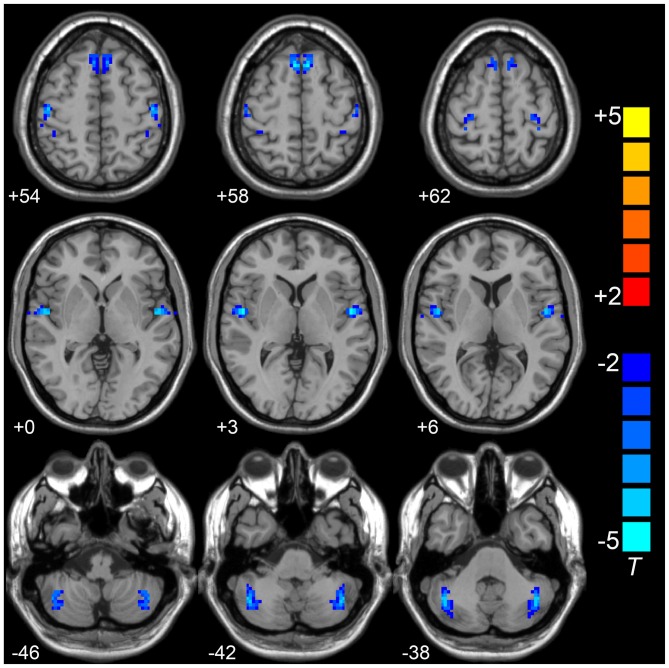
**Compared to HCs group,VMHC in superior frontal gyrus (SFG), superior temporal gyrus (STG), posterior cerebellar lobe (CePL), postcentral and precentral gyri was observed in LOD.** Blue color represents reduced VMHC value in LOD. The numbers at the down left of each image refer to the z-coordinates in Montreal Neurological Institute template. The threshold was set at a corrected *P* < 0.001 (corrected with *P* < 0.01 for each voxel and cluster volume ≥55 voxels) and the *t*-score bar is present at the right-side. Notes: HCs, Healthy Controls; VMHC, Voxel-Mirrored Homotopic Connectivity; LOD, Late-Onset Depression.

**Figure 2:**
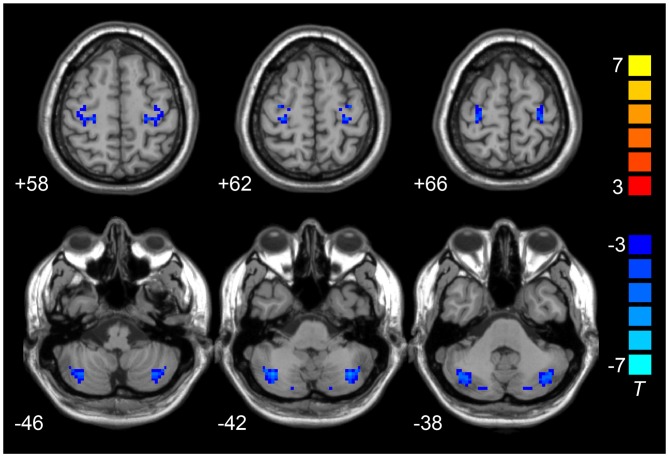
**Results of split-half sample validation.** Comparisons of VMHC were analyzed between 16 LOD and 18 HCs. Blue color represents reduced VMHC value in LOD. The numbers at the down left of each image refer to the *z*-coordinates in Montreal Neurological Institute template. The threshold was set at a corrected *P* < 0.001 (corrected with *P* < 0.01 for each voxel and cluster volume ≥55 voxels) and the *t*-score bar is present at the right-side. Notes: HCs, Healthy Controls; VMHC, Voxel-Mirrored Homotopic Connectivity; LOD, Late-Onset Depression.

Compared to the HCs, the LOD group showed significant low interhemispheric homotopic coordination in the superior frontal gyrus (SFG), superior temporal gyrus (STG), posterior cerebellar lobe (CePL) and postcentral and precentral gyri (Table 2, Figure 1). No brain regions with higher VMHC were detected between two groups. By split-half sample validation, the regions of lower VMHC were confirmed in the CePL, postcentral and precentral gyri (Figure 2), which were highly in line with the results from the full sample.

**Table 2:** **The decreased regional VMHC in LOD group relative to HCs**.

Brain regions	BA	Voxel number	Coordinates MNI			*T*-score
			*X*	*Y*	*Z*
LOD < HCs:
Superior frontal gyrus	10	100	6	21	57	−5.34
Superior temporal gyrus	22	74	51	−6	3	−5.46
Cerebellum posterior lobe	/	117	45	−63	−36	−5.13
Postcentral gyrus	2/3	65	27	−24	66	−4.95
Precentral gyrus	4	56	17	−29	73	−5.07

*Abbreviations: VHMC, Voxel-Mirrored Homotopic Connectivity; LOD, Late-Onset Depression; HCs, Healthy Controls; BA, Brodmann Area; MNI coordinates, Montreal Neurological Institute coordinates of the peak voxel; T, the statistical value of the peak intensity voxel; 1 voxel = 3 mm × 3 mm × 3 mm. The MNI coordinates of peak voxel are symmetrical as the VMHC computation bases on the symmetrical template*.

### Neuro-Behavioral Relevancy Analysis Between VMHC and Neuropsychological Variables

**Figure 3:**
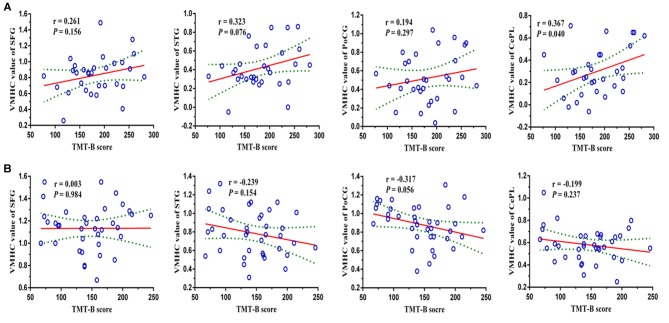
**Pearson’s correlation analysis revealed that the reduced VMHC strength in bilateral CePL was positively correlated with the performance of TMT-B in LOD (*r* = 0.367, *P* = 0.040).** Notes: VMHC, Voxel-Mirrored Homotopic Connectivity; SFG, Superior Frontal Gyrus; STG, Superior Temporal Gyrus; PoCG, Postcentral Gyrus; CePL, Posterior Cerebellar Lobe; TMT-B, Trail Making Test B; LOD, Late-Onset Depression. The figures of row **(A,B)** represent the relationships (TMT-B and regional VMHC values) in LOD and HCs, respectively.

Pearson’s correlation analysis suggested that the reduced VMHC strength in bilateral CePL was positively correlated with the performance of TMT-B in LOD patients (*r* = 0.367, *P* = 0.040). No significant relationship was confirmed in the LOD or HC groups between the VMHC in other brain regions and the neuropsychological variations (Figure 3).

### The Diagnostic Performance of Altered VMHC in Differentiating LOD from HCs

**Figure 4:**
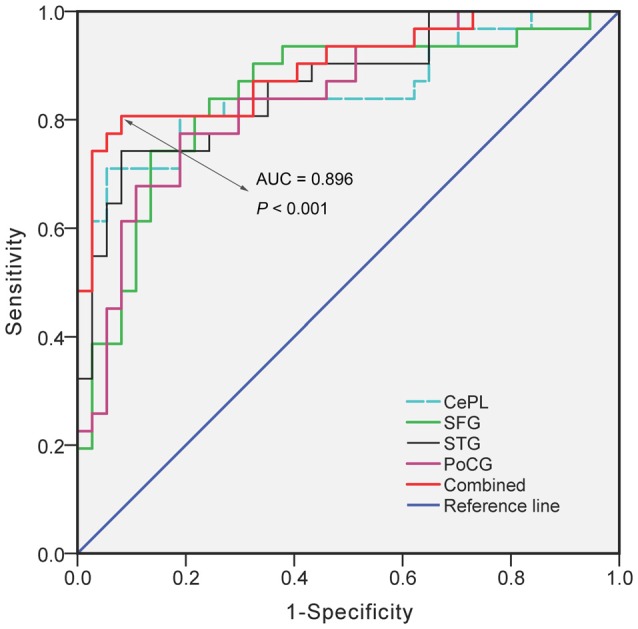
**The diagnostic performance of disrupted VMHC in differentiating LOD from HCs.** Notes: VMHC, Voxel-Mirrored Homotopic Connectivity; LOD, Late-Onset Depression; HCs, Healthy Controls; CePL, Posterior Cerebellar Lobe; SFG, Superior Frontal Gyrus; STG, Superior Temporal Gyrus; PoCG, Postcentral gyrus; AUC, Area Under Curve.

ROC analysis demonstrated that the regional VMHC changes of CePL (AUC = 0.853, *P* < 0.001), SFG (AUC = 0.845, *P* < 0.001), STG (AUC = 0.864, *P* < 0.001) and postcentral gyrus (PoCG) (AUC = 0.840, *P* < 0.001) exhibited good performance in distinguishing LOD patients from HCs. Moreover, when the combined effects of these regional changes were taken into account, the outstanding differentiated ability (AUC = 0.896, *P* < 0.001) was achieved with balanced sensitivity (84%) and specificity (73%; Figure 4).

## Discussion

In the present study, we first examined the differences of resting-state voxel-wise VMHC and the possible interaction with cognitive performance in LOD patients and HCs. For LOD patients, significantly decreased VMHC were observed in bilateral STG, post- and precentral gyri, SFG and CePL as compared with HCs. Compatible with previous studies, the overall cognitive performance (as assessed by MMSE) or specific domains of cognition (i.e., TMT, SDMT) significantly declined in healthy subjects (Richard-Devantoy et al., 2013; Sachs-Ericsson et al., 2013). Furthermore, the novel pattern of positive correlation between disrupted homotopic coordination (decreased VMHC) in CePL and total completion time on TMT-B was confirmed in this study. Importantly, these altered regional VMHC showed good performance in differentiating LOD patients from HCs.

Evidence from neuroimaging studies concerning STG and post/precentral gyri have yielded conflicting results. A regional homogeneity study reported significantly increased spontaneous neural activity in both the left STG and right PoCG (Liu et al., 2012), whereas other research demonstrated the decreased activity coherence in the PoCG in elderly depressed patients (Ma et al., 2013). A recent coordinate-based meta-analysis highlighted increased activation in the left precentral gyrus and right superior temporal with reduced activity in the right precentral gyrus during working memory tasks (Wang et al., 2015a). Furthermore, increased activation was observed in the right precentral gyrus that underlies dysfunctional cognitive processing during semantic verbal fluency in depression (Backes et al., 2014). Additionally, the decreased interhemispheric coordination in STG was found in treatment-resistant depression (Guo et al., 2013). STG and post/precentral gyri accounted for language processing and motor supplementary, respectively and were critical for executive and social function (Kana et al., 2015; Roux et al., 2015; Upadhyay et al., 2016). The reduced VMHC of STG and post/precentral gyri in the present study may disrupt the integration of bilateral brain function when dealing with an emotional event or cognitive task. As a core component of DMN, SFG is involved in the process of emotion and cognition, including self-referential processing, learning function, episodic memory and working memory processing (Bai et al., 2011; Coutinho et al., 2016). The atrophy and disrupted white matter integrity of SFG was proposed to be a marker of disease severity and impending cognitive decline in late-life depression (Reppermund et al., 2014; Boccia et al., 2015). Furthermore, the volumetric alteration in SFG was correlated with numerical daily function, including working memory and abstract reasoning (Benavides-Varela et al., 2015). Our previous work also demonstrated that the abnormal intrinsic activity in SFG was related to cognitive dysfunction in LOD (Yue et al., 2015). Specifically, in the present study, the disrupted interhemispheric coordination in bilateral SFG may be representative of poor cognitive control regulating the default mode in the processing of emotional reaction, further supporting the viewpoint that impairment in the neural network led to an increased susceptibility to early cognitive deficit in LOD. The notable effect of altered regional VMHC (STG, postcentral gyri, SFG and CePL) in differentiating LOD was also primarily confirmed in this study. The distinct performance identified in the ROC indicated that the combined VMHC change of several brain regions rather than that of a single region can be more accurate in distinguishing the LOD patients from HCs. Those synthetical results further suggest that the neural activities in those bilateral brain regions were desynchronized in LOD patients with cognitive impairment. In sum, the disrupted homotopic connectivity in these regions may assist in the diagnostic decision and early intervention of LOD as well as the identification of potential conversion to dementia.

Intriguingly, the novel positive relationship between reduced VHMC in bilateral cerebellum posterior lobes and TMT-B score was detected in the present study. Generally, completion time on TMT-B is considered to constitute a behavioral estimate of the capacity of cognitive flexibility, attributed to the function of frontal lobes. Using voxel-based lesion-behavior mapping, the right hemispheric frontal lesions were reported to be associated with TMT-B score (Kopp et al., 2015). Traditionally, the cerebellum is thought to account for body balance and sensomotor function. The cerebellum has anatomically reciprocal connections with the cerebral cortex and limbic regions involved in complex cognitive operation and emotional processing (Stoodley and Schmahmann, 2009, 2010). The volumetric cerebellum reduction was observed in depression (Grieve et al., 2013), and the aberrant cortico-cerebellar functional networks were associated with impaired episodic memory and emotional regulation (Balsters et al., 2014). Additionally, Lai and Wu (2014) reported that the weaker interhemispheric coordination between bilateral CePL probably represented the disturbed function of the emotional and cognitive process. It should be noted that no evidence was proposed to the linkage between altered VMHC within bilateral cerebellum and cognitive impairment in LOD. The present results indicated that the depressive state might influence the interhemispheric information communication for cognitive processing, and the neural circuitry modulating cognition was pivotal as scaffolding to mediate adverse emotion effect. The underlying mechanisms of these abnormal coordination across bilateral hemispheres in resting state are still unclear and deserve further investigation.

There were methodological considerations and limitations in the present study that must be acknowledged. First, by lacking follow-up data, we couldn’t determine the discrepancy of VMHC with time-related trajectory between LOD patients and HCs across the aging process. Cortical atrophy is also a potential factor that might impact the homotopic coordination in our study. Second, the education level between the LOD patients and HCs in this study was not well matched, even though we controlled the bias by the statistical method; future work with homogeneous participants would help verify whether the findings are common. Finally, the results of this study only stem from the single modality of rs-fMRI; it should benefit by utilizing optimized analytical strategies and multiple imaging modalities (e.g., white matter fiber-tracking) to understand the structural substrate underlying the VMHC alteration. Future works with longitudinal design, data analyses of multiple modalities imaging, and well-matched subjects are warranted to replicate and validate these findings.

Collectively, our study was designed as a pragmatic work to explore the potential alterations of integrative processing and information communication in LOD with cognitive deficit. The relationship between abnormal VMHC strength and cognitive dysfunction was first explored in LOD. It will likely shed further light on the permanency of neuropathological traits in the deterioration of dementia. The results of differentiated regional VMHC changes indicate that the neuroimaging-guided differential diagnosis could serve as promising neural targets for personalized diagnosis and optimal treatment in future clinical practice.

## Author Contributions

ZH recruited the participants, collected the data, performed data analyses and prepared the manuscript. XS helped with data analyses and reviewed the manuscript. YS helped with patients enrollment and data analyses. YY designed the study and supervised preparation of the manuscript.

## Conflict of Interest Statement

The authors declare that the research was conducted in the absence of any commercial or financial relationships that could be construed as a potential conflict of interest. The handling Editor declared a shared affiliation, though no other collaboration, with one of the authors XS and states that the process nevertheless met the standards of a fair and objective review.

## Footnotes

^1^http://www.restfmri.net

^2^http://www.fil.ion.ucl.ac.uk/spm

^3^http://afni.nimh.nih.gov/pub/dist/doc/manual/AlphaSim.pdf
